# TLR4-mediated chronic neuroinflammation has no effect on tangle pathology in a tauopathy mouse model

**DOI:** 10.3389/fnagi.2024.1468602

**Published:** 2024-10-21

**Authors:** Neha Basheer, Muhammad Khalid Muhammadi, Carlos Leandro Freites, Martin Avila, Miraj Ud Din Momand, Natalia Hryntsova, Tomas Smolek, Stanislav Katina, Norbert Zilka

**Affiliations:** ^1^Institute of Neuroimmunology, Slovak Academy of Sciences, Bratislava, Slovakia; ^2^Institute of Histology and Embryology of Mendoza (IHEM), National University of Cuyo, National Scientific and Technical Research Council (CONICET), Mendoza, Argentina; ^3^Institute of Mathematics and Statistics, Faculty of Science, Masaryk University, Brno, Czechia

**Keywords:** neuroinflammation, tau, lipopolysaccharide, phosphorylation, microglia

## Abstract

**Introduction:**

Alzheimer’s disease (AD) is marked by the accumulation of fibrillary aggregates composed of pathological tau protein. Although neuroinflammation is frequently observed in conjunction with tau pathology, current preclinical evidence does not sufficiently establish a direct causal role in tau tangle formation. This study aimed to evaluate whether chronic Toll-like receptor 4 (TLR4) stimulation, induced by a high dose of lipopolysaccharide (LPS, 5 mg/kg), exacerbates neurofibrillary tangle (NFT) pathology in a transgenic mouse model of tauopathy that expresses human truncated 151-391/3R tau, an early feature of sporadic AD.

**Methods:**

We utilized a transgenic mouse model of tauopathy subjected to chronic TLR4 stimulation via weekly intraperitoneal injections of LPS over nine consecutive weeks. Neurofibrillary tangle formation, microglial activation, and tau hyperphosphorylation in the brainstem and hippocampus were assessed through immunohistochemistry, immunofluorescence, and detailed morphometric analysis of microglia.

**Results:**

Chronic LPS treatment led to a significant increase in the number of Iba-1^+^ microglia in the LPS-treated group compared to the sham group (*p* < 0.0001). Notably, there was a 1.5- to 1.7-fold increase in microglia per tangle-bearing neuron in the LPS-treated group. These microglia exhibited a reactive yet exhausted phenotype, characterized by a significant reduction in cell area (*p* < 0.0001) without significant changes in other morphometric parameters, such as perimeter, circumference, solidity, aspect ratio, or arborization degree. Despite extensive microglial activation, there was no observed reduction in tau hyperphosphorylation or a decrease in tangle formation in the brainstem, where pathology predominantly develops in this model.

**Discussion:**

These findings suggest that chronic TLR4 stimulation in tau-transgenic mice results in significant microglial activation but does not influence tau tangle formation. This underscores the complexity of the relationship between neuroinflammation and tau pathology, indicating that additional mechanisms may be required for neuroinflammation to directly contribute to tau tangle formation.

## 1 Introduction

Alzheimer’s disease (AD) and related tauopathies are characterized by the stereotypical accumulation of fibrillary aggregates primarily composed of hyperphosphorylated and truncated tau (Novak et al., 1993; Grundke-Iqbal et al., 1986; Wang et al., 2013). The pathobiology of tauopathies consistently reveals an association with neuroinflammation, evidenced by the frequent presence of reactive microglia near tau inclusions (Bellucci et al., 2011; Ishizawa and Dickson, 2001; Sasaki et al., 2008; Serrano-Pozo et al., 2013). Neuroinflammation follows a similar temporal course to tau pathology (Eikelenboom et al., 2010; Jaworski et al., 2011; López-González et al., 2015; Yoshiyama et al., 2007; van Olst et al., 2020), suggesting a tight interplay between these processes.

Microglia, as the primary responders to neuronal distress, undergo substantial adaptive phenotypic and functional alterations during the course of pathology (Sobue et al., 2021; Grubman et al., 2019; Mathys et al., 2019; Zhou et al., 2020). While these alterations initially serve a protective role (Keren-Shaul et al., 2017), they can become deleterious (Liddelow et al., 2017) after reaching a “tipping-point” (Simons et al., 2023). In the context of tau pathology, microglial reactivity is thought to amplify tau hyperphosphorylation and the subsequent formation of tangles. Conversely, existing pathological inclusions may induce further microglial reactivity, suggesting a fatal vicious cycle between tau pathology and neuroinflammation (Laurent et al., 2018; Nilson et al., 2017; Leyns and Holtzman, 2017; Zilka et al., 2012). Several studies have investigated this hypothesis, predominantly examining acute effects through modeling neuroinflammation with lipopolysaccharide (LPS)-mediated TLR4 stimulation in wild-type animals that do not develop tangle pathology. In transgenic models, the fewer chronic studies often focus exclusively on levels of tau hyperphosphorylation at different epitopes rather than quantifying tangle pathology (Catorce and Gevorkian, 2016; Batista et al., 2019; Barron et al., 2017). Furthermore, the use of low doses of LPS in chronic inflammation setups is not justified, as rodents are much less sensitive to endotoxin than humans (Seok et al., 2013). Common experimental doses range from 0.1 to 20 mg/kg (Catorce and Gevorkian, 2016), but the studies investigating effect of chronic LPS administration employed lower doses of LPS (<1 mg/kg; Catorce and Gevorkian, 2016; Batista et al., 2019; Barron et al., 2017). Humans experience a 2-to-3-fold increase in circulating plasma levels of LPS with age (Sanchez-Tapia et al., 2023), and an even larger increase (approximately 2-to-10-fold) is observed in other chronic inflammatory disorders (Cani et al., 2007; Mohammad and Thiemermann, 2020; Beam et al., 2021; Jayashree et al., 2014; Wiedermann et al., 1999), significantly raising the risk of developing AD. Although LPS is rapidly cleared from the bloodstream, its tissue distribution can prolong the inflammatory response. However, the response to lower doses used in chronic inflammation setups in tau transgenic mice is not sustained long enough to be relevant to the chronic neuroinflammation observed in AD.

This study, therefore, focused on elucidating the impact of TLR4 stimulation mediated by LPS on tangle formation. We used higher dose of LPS that does not induce sepsis but is relevant to human conditions. We employed a tau-transgenic mouse model expressing truncated tau, which can develop full-blown tangle pathology that fulfils all criteria of human tangle pathology. We performed semiautomated quantification of the tangle-bearing neurons and microglia, followed by microglial morphometry to highlight their interaction.

## 2 Materials and methods

### 2.1 Animals

Adult C57BL/6J and tau-transgenic R3m4 mice expressing human truncated tau protein (3R tau, aa151-391) under the Thy1 promoter were utilized in this study. The expression of this transgene has been demonstrated to induce the formation of tau tangles in rats, making it the sole rat model that exhibits progressive, age-dependent neurofibrillary degeneration in the cortical brain regions (Filipcik et al., 2012; Koson et al., 2008; McMurray et al., 2021). R3m4 mice were genotyped by PCR of DNA extracted from tails (Zimova et al., 2016). All animals were housed under standard laboratory conditions, resided in plastic cages with four animals per cage, and were provided with *ad libitum* access to food and water. A diurnal lighting schedule of 12-h light/dark cycles with a light phase beginning at 7:00 a.m. was maintained, and the temperature (22 ± 2°C) and humidity (55 ± 10%) were controlled. All the ethical considerations were strictly followed, and all the experiments adhered to international and institutional animal care guidelines.

### 2.2 Methods of randomization, sex proportionality, and allocation concealment

Considering the observed variability in the inflammatory response following LPS challenge, attributed to factors such as the specific LPS serotype, mouse strain, injection site, administered LPS dose, and duration before the animals were sacrificed (Catorce and Gevorkian, 2016), our initial objective was to determine the neuroinflammatory efficacy of the chosen high dose of LPS (5 mg/kg) in wild-type C57BL/6J mice (*n* = 3, female, 2 months old, 20–23 g). To further investigate the effects of chronic TLR4 stimulation via LPS on tauopathy, a total of 20, age- and weight-matched, R3m4 mice were included in this study and randomly divided into two groups: a sham group (*n* = 10, 5 males and 5 females) and an LPS-treated group (*n* = 10, 5 males and 5 females). The animals were documented by their IDs and were randomly assigned to different cages at the time of birth considering equal representation of sex in each group. The individuals performing the randomization process were unaware of the grouping, ensuring blinding from the start. Mice from the same experimental group were housed together to ensure consistent conditions and minimize confounding variables. We maintained accurate records throughout the study to track the progress and outcomes of the individual mice.

### 2.3 LPS administration

**Figure 1:**
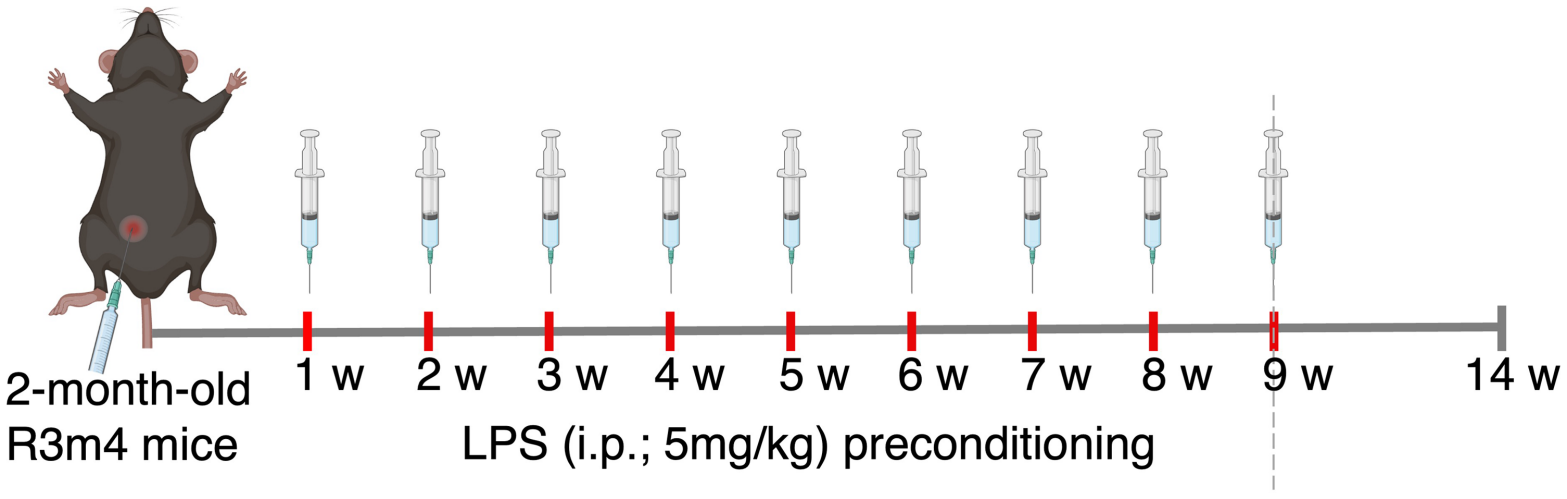
Schematics of the experimental design.

LPS from *E. coli* O26:B6 (Sigma–Aldrich, USA) under sterile conditions was reconstituted in 1× phosphate-buffered saline (1 × PBS) to the concentration of 1 mg/ml. For the purpose of testing dose response, corresponding to a high dose of 5 mg/kg body weight, a total volume of 120 μl was injected intraperitoneally (i.p.) into two groups of 2-month-old C57BL/6 mice (*n* = 3/group; females). One group was sacrificed 24 h after the LPS injection, and the other was sacrificed after 7 days. The sham group received PBS injections in the same manner. To further investigate the effects of chronic TLR4 stimulation via LPS on tauopathy, 2-month-old tau-transgenic R3m4 mice were injected with either the tested LPS dose or PBS weekly for 9 consecutive weeks. Throughout the study, the body weight and general health status of each mouse were recorded on a weekly basis, and the mice were sacrificed 4 weeks after the last injection, to exclude the acute inflammatory effects of the last LPS insult (Figure 1).

### 2.4 Immunohistochemistry and immunofluorescence

Immunohistochemistry (IHC) and immunofluorescence (IF) was performed on 40 μm thick coronal free-floating sections, as described in detail previously (Mate et al., 2022). For AT8-positive neurons in the hippocampus, we stained every sixth, for a total of six sections/animal (*n* = 8/group), spaced ~240 μm apart. For tau tangle counts in the brainstem, we stained every tenth section, for a total of eight sections per animal (*n* = 8/group), spaced ~400 μm apart. For microglial morphometry, we chose two sections from the brainstem ~400 μm apart (*n* = 3/group). All the sections were incubated overnight at 4°C with primary antibodies (Table 1), followed by incubation with the appropriate biotin-conjugated secondary antibodies and visualized using a Vectastain ABC Kit (Vector Laboratories, CA, United States) for IHC or with the appropriate secondary Alexa Fluor antibodies for IF. Finally, the sections were mounted, and the IHC-stained sections were evaluated using an Olympus BX51 microscope equipped with a Nikon digital camera (DS-Fi3) and the software NIS-Elements AR 5.02.01, whereas the IF-stained sections were examined with an LSM 710 laser scanning confocal microscope (Carl Zeiss, Jena, Germany).

**Table 1:** List of primary and secondary antibodies utilized for IHC.

Antibody	Host	Dilution	Source
Anti-human pSer202/pThr205 tau (AT8)	Mouse	1:1000	Thermo Scientific (Rockford, IL, USA) #MN1020
DC217 (pThr217)	Mouse	1:1000	AXON Neuroscience SE (Bratislava, Slovak Republic)
Anti-Iba-1 (Iba-1C-terminal sequence)	Rabbit	1:1000	FUJIFILM Wako Pure Chemical Corporation (Richmond, VA, USA) #019-19741
Anti-GFAP	Rabbit	1:2000	Abcam (Cambridge, United Kingdom) #ab7260
Anti-mouse IgG antibody (H + L), biotinylated	Horse	1:1000	Vector laboratories #BA-2000-1.5
Anti-rabbit IgG antibody (H + L), biotinylated	Goat	1:1000	Vector laboratories #BP-9100-50
Anti-CD68 antibody [EPR23917-164]	Rabbit	1:250	abcam #ab283654

### 2.5 Quantification of microglia, tau tangles, and tau phosphorylation: general analysis workflow

Following image acquisition, analysis was conducted on a MacBook Air with the Apple M1 chip running macOS Sonoma version 14.0 using QuPath (v0.4.3; https://qupath.github.io/; Bankhead et al., 2017). Six hippocampal and eight brainstem slices per animal (*n* = 8/group) were selected for quantification in separate projects within QuPath. Colour deconvolution was applied to whole-slide images to separate the stains into three channels: the VIP chromogen, methyl green, and a residual channel. To exclude potential false positives and eliminate folded tissue areas, manual annotations were created around tissue edges. To quantify Iba-1+ monocyte/macrophage/microglia and tau tangles, superpixel analysis was employed. QuPath systematically processes all the pixels within each image, grouping adjacent similar pixels into superpixels of a predetermined size. Pixel similarity is determined based on red–green–blue (RGB) values, reflecting colour and shade. In an annotated region, DoG (Difference of Gaussians) superpixel creator feature was applied with the following parameters: downsample factor = 0.1, Gaussian sigma = 0.5, minimum intensity threshold = 2, maximum intensity threshold = 2, and noise threshold = 0.5. After superpixel grouping, intensity features were set for the VIP channel. Using the brush tool, regions were labelled as ‘positive’ or ‘negative’. The object classifier feature was executed, optimized, and saved. The classifier was next loaded for application to tissue sections across the project. The resulting number of detections provided the count of observed tangles and microglia in each slice. A workflow with selected commands from both analyses was saved as a script and executed for the entire project, ensuring consistency in the analysis across individual projects.

Signal intensity of tau phosphorylation from hippocampal and brainstem slices was quantified using NIH ImageJ software (Fiji version). The images were converted to an 8-bit grayscale format. The scale was calibrated using the scale bar, and regions of interest (ROIs) were selected with the Rectangle or Freehand selection tool, managed through the ROI Manager. Signal intensity within each ROI was measured by obtaining the mean gray value, and background intensity was determined by measuring an unstained area. Corrected signal intensity was calculated by subtracting the background intensity from the signal intensity of the ROIs. Measurement results were exported as CSV files for further analysis. For batch processing of multiple images, a macro was recorded to automate the process, including steps for opening images, converting to 8-bit, defining ROIs, measuring intensities, and saving results.

### 2.6 Microgial morphological assessment: image acquisition, preprocessing, shape descriptors, and Sholl analysis

The morphological variables of the microglial cell population were evaluated by a condition-blind observer using the FIJI (Young and Morrison, 2018). Microglia were randomly selected from the Pontine Reticular Nucleus (PRN), where the AT8 signal was evident. Three z-stack images per PRN were acquired. Each original z-stack was processed in a semiautomated way with the same macro on FIJI. First, a subtract background filter was applied to maintain the quality of the image. The image was subsequently converted into an 8-bit grayscale image, and an Unsharp Mask filter was applied to sharpen the Iba-1 signal and cell processes. Next, a despeckle step was performed to eliminate salt-and-pepper noise. The images were then auto-thresholded to create a binary mask for each image. Once these binary masks were created, with the BioVoxxel Toolbox plug-in, a series of binary steps were performed to join the cell processes: the Close, Dilate, Close and Erode functions were applied. Each thresholded image was inspected to ensure that accurate reconstruction was performed by the software. Otherwise, the paintbrush tool was used to join evident ramifications of the cell. Finally, an analysis of particle function was performed to “clean” the final binary mask, allowing only the cells of interest to be subjected to further analysis.

Individualized microglial binary masks were randomly selected by the FIJI using the ROI Manager tool. Once the microglial mask was selected, each morphological parameter of interest was calculated by the Measure function. The area, perimeter, aspect ratio, circularity, and solidity were calculated for each random cell line as previously described for microglia (Fernandez-Arjona et al., 2017). At least 3 cells per image were quantified, generating a total of 33–35 cells per animal, and more than 100 cells were analysed per condition. The same individualized microglial binary masks used for the shape descriptors were utilized to perform Sholl analysis via the Neuroanatomy plug-in on FIJI. The starting radius was defined at 10 pixels to exclude the soma from the analysis, while the step size was set to three pixels. The length of the concentric circle drawing was set to 250 pixels, enabling us to ensure that all the processes of each different cell were counted. The data from each table generated were copied, and the mean number of intersections per radius per condition was used to generate the final Sholl plot (# of intersections vs. distance from the soma).

### 2.7 Statistics

Data processing and statistical analyses were performed in the R programming environment version 4.3.2 (R Development Core Team, 2023). All alternative hypotheses were two-sided, and statistical tests were performed at a significance level equal to 0.05. All the empirical confidence intervals (CIs) used were Wald type, 95%, and two-sided. For each treatment (LPS and Sham) and animal, the data were winsorized using the Tukey interquartile (Tukey, 1962) approach (due to the presence of outliers). The null hypothesis that the mean difference between the LPS and sham treatment is equal to zero, H_0_: μ_controls_ − μ_LPS_ = 0, was tested against the alternative hypothesis that the mean difference between the LPS and sham treatment is not equal to zero, H_1_: μ_controls_ − μ_LPS_ ≠ 0, by two-sample Wald statistics (*t*-statistics) with Satterthwaite error degrees of freedom taken from the mixed-effect linear regression model (MELRM; Pinheiro and Bates, 2006) calculated by the profile method with proportional weights. The MELRM model was used in the following form: variable ∼ treatment + (1|animal), where the variables were AT8-positive neurons (%), AT8-positive NFTs, DC217-positive NFTs, and Iba-1-positive cells; all the morphological variables (area (pixels), perimeter (pixels), circularity, solidity, and aspect ratio); and area under the curve (AUC, the curve defined as the number of intersections (ramifications) on the basis of the distance from the center of the soma (body cell)) as a part of the other morphological analysis. The treatment (coded as LPS and Sham) was the fixed main effect, and the animal was the random effect (random intercept). Applying this MELRM to repeated observations (here, e.g., 8 slices), the repeated observations per animal are taken into account individually; thus, the variability within and between animals is correctly estimated. The results are summarized numerically by the mean difference (sham minus LPS), lower and upper bounds of 95% CIs for the mean difference, and *p* value and graphically by boxplots. The repeated measures correlation coefficient was calculated from a linear regression model (Bakdash and Marusich, 2017) with the animal as a fixed effect (allowing for different intercepts for each animal), and the common slope was calculated separately for each treatment. The data were visualized as scatterplots with regression lines for each animal. The value of the coefficient does not depend on which variable is taken as dependent or independent (in this respect, it is symmetric). AT8-positive NFTs were compared with DC217-positive NFTs, AT8-positive NFTs were compared with Iba-1-positive cells, and DC217-positive NFTs were compared with Iba-1-positive cells. The results are summarized numerically by correlation coefficient, lower and upper bound of 95% CIs for correlation coefficient (back-transformed from Fisher *Z*-transformation) and *p* value.

## 3 Results

### 3.1 Administration of a single high dose of LPS induced neuroinflammation

**Figure 2:**
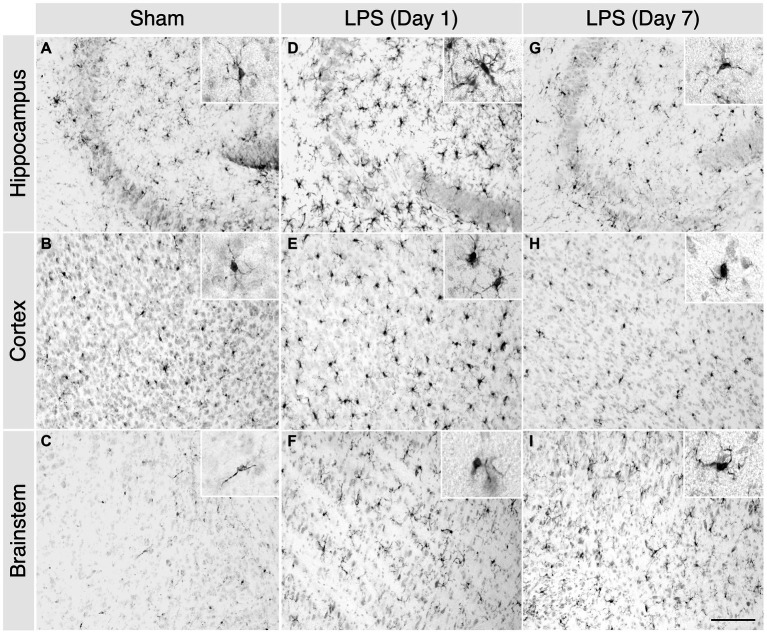
A single high dose of LPS induced microgliosis in WT mice. **(A–C)** In the sham group, 1 day post i.p. PBS administration, homeostatic Iba-1^+^ cells were detected in the hippocampus, cortex, and brainstem. **(D–F)** However, in the LPS group, an enhanced number of reactive Iba-1^+^ cells were detected in the hippocampus, cortex, and brainstem. **(G–I)** In the LPS group 7-days post administration, the characteristic reactive morphology of microglia was also evident in the hippocampus, cortex, and brainstem (2-month-old C57BL/6 mice, *n* = 3/group). Magnification, 20×; inset, 40×. Scale bar, 100 μm.

**Figure 3:**
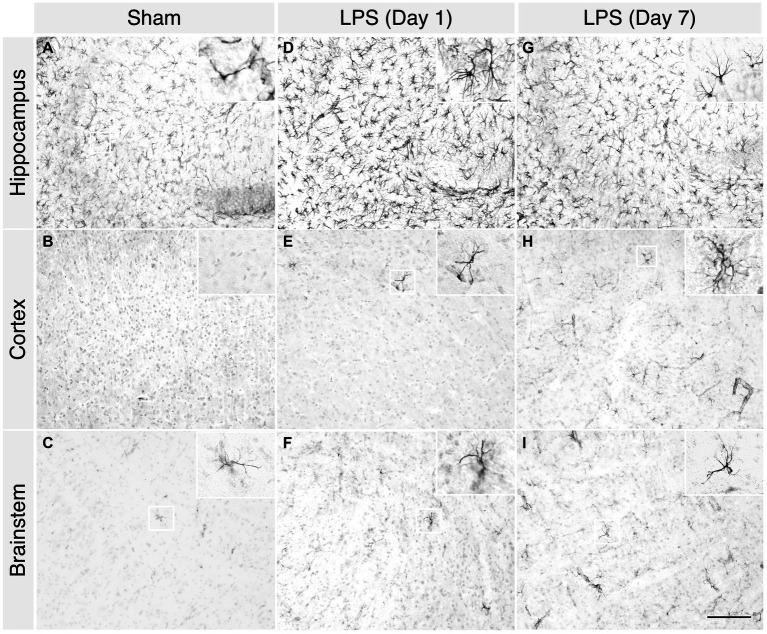
A single high dose of LPS induced astrogliosis in WT mice. **(A–C)** In the sham group, 1 day post i.p. PBS administration, sparse GFAP^+^ astrocytes were detected in the hippocampus, cortex, and brainstem. **(D–F)** However, in the LPS group, an increased number of GFAP^+^ astrocytes were detected in the hippocampus, cortex, and brainstem. **(G–I)** In the LPS group, on 7 days post administration, the characteristic bushy morphology of astrocytes was evident in the hippocampus, cortex, and brainstem (2-month-old C57BL/6 mice, *n* = 3/group). Magnification, 20×; inset, 40×. Scale bar, 100 μm.

LPS-treated mice exhibited classic signs of sickness behaviours, including decreased locomotion, a hunched posture, and anorexia. We observed a noticeable increase in microglial proliferation and heightened responsiveness in the hippocampus, brainstem, and cortex at 1 day post LPS administration (Figures 2D–F). Phenotypically, there was an augmentation in microglial process retraction, manifesting as bipolar or unipolar configurations with elongated, swollen processes and cell bodies, characteristic of microgliosis. In contrast, homeostatic microglia in the sham group persisted at baseline, maintaining their characteristic phenotype (Figures 2A–C). By day 7th post LPS administration, we observed an apparent decrease in the number of microglia, although the microglia still displayed signs of microgliosis (Figures 2G–I). Similarly, with upregulated expression of glial fibrillary acidic protein (GFAP), we observed the presence of astrocytes characterized by hypertrophic cell bodies and predominantly main processes, in the hippocampus, cortex and brainstem on day 1 post administration (Figures 3D–F). In contrast, the astrocytes in the sham group exhibited a homeostatic morphology (Figures 3A–C). Notably, a partial resolution of the phenotype in terms of the number of astrocytes was observed on day 7 (Figures 3G–I).

### 3.2 LPS-mediated chronic TLR4 stimulation led to an increase in Iba-1 positive monocyte/macrophage/microglia and sustained changes in microglial morphology

**Figure 4:**
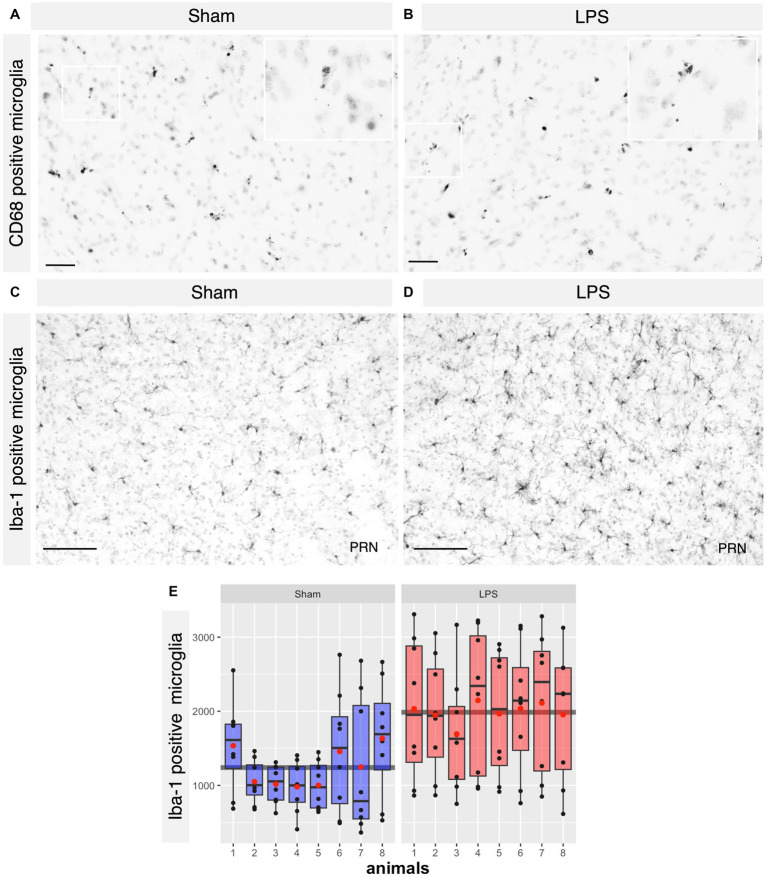
No difference in the CD68^+^ microglia where significant proliferation of microglia was observed in the LPS-treated group. **(A,B)** Representative IHC sections of the brainstem probed for CD68 in both the sham and LPS-treated groups. **(C,D)** Representative IHC sections of the brainstem probed for Iba-1 in both the sham and LPS-treated groups. **(E)** A significant increase in the number of Iba-1^+^ cells was observed in the LPS-treated group compared to the sham-treated R3m4 mice (*p* < 0.0001). Boxes indicate medians and quartiles for Iba-1^+^ cells count per animal; whiskers indicate quartiles ± 1.5× IQR. The black dots indicate the microglial count per slice. The red dots indicate the arithmetic mean. Magnification, 20×; inset, 40×. Scale bar, 50 μm, 100 μm.

**Figure 5:**
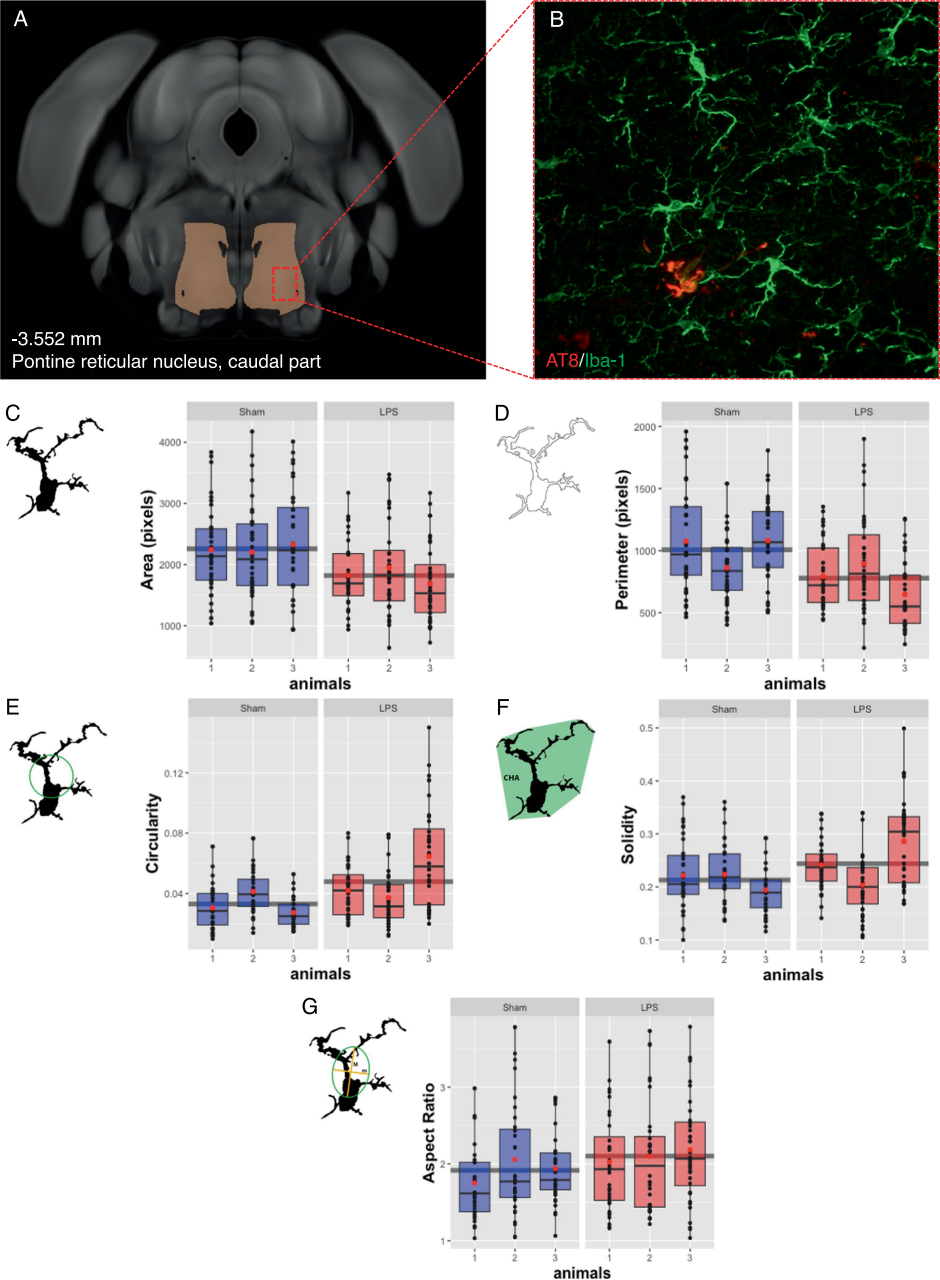
Subtle changes in microglial morphology were observed after LPS-mediated chronic TLR4 stimulation. **(A)** Schematic illustration of the region under investigation for morphological assessment of Iba-1^+^ cells. **(B)** Representative image of the IHC. **(C)** The area changed significantly in the LPS-treated animals compared to the sham animals (*p* < 0.0001). **(D)** The perimeter, **(E)** the circularity, **(F)** the solidity, and **(G)** the aspect ratio did not significantly differ. Boxes indicate medians and quartiles for morphometric parameters per animal; whiskers indicate quartiles ± 1.5× IQR. The black dots indicate the microglial count per slice. The red dots indicate the arithmetic mean.

**Figure 6:**
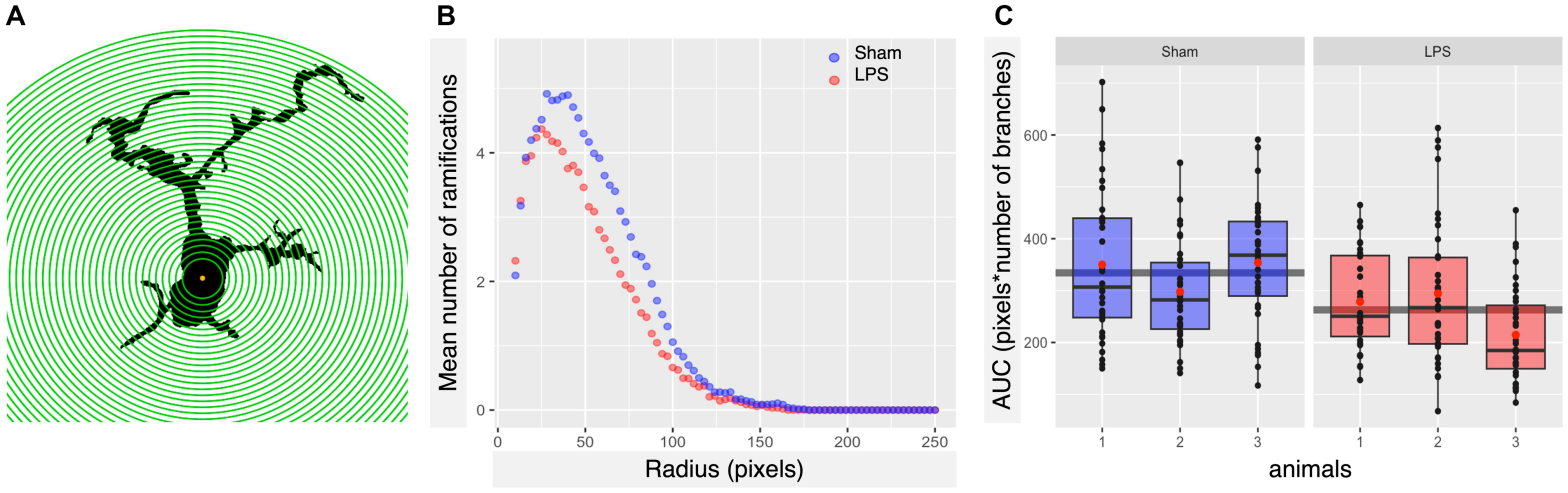
Subtle LPS associated alterations in the arborization state of microglia were observed in the brainstem. **(A)** Schematic illustration of Iba-1^+^ cell masks, with concentric circles utilized for Sholl analysis. **(B)** Sholl plot per group showing the average number of microglial cell branch intersections per 3-pixel steps from the cell soma. **(C)** LPS preconditioning appears to induce a slight decrease in the number of ramifications of microglia in R3m4 mice; however, the Sholl-derived area under the curve (AUC) analysis did not reveal a statistically significant reduction in the number of microglial cell branches (*p* = 0.078). Boxes indicate medians and quartiles for AUCs per animal; whiskers indicate quartiles ± 1.5× IQR. The black dots indicate individual microglia. The red dots indicate the arithmetic mean.

Based on tolerability and observed glial reactivity in wild-type mice, we investigated the effect of LPS-mediated chronic TLR4 stimulation in tau transgenic R3m4 mice. First, we quantified the number of microglia within the brainstem, where tau neurofibrillary pathology originally develops (Zimova et al., 2016). We found no apparent difference in CD68+ cells (Figures 4A,B). However, we observed a notable increase in the abundance of microglia in LPS-treated group compared to sham (mean difference, −746.13; 95% CI, −1007.618, −484.632; *p* < 0.0001; Figures 4C–E). Next, we conducted microglial morphometry in the pontine reticular nucleus (PRN) which is heavily burdened with NFT pathology (Figures 5A,B; Zimova et al., 2016). Microglia in the LPS-treated animals exhibited a statistically significant decrease in cell area (mean difference 437.79; 95% CI 244.309, 631.276; *p* < 0.0001; Figure 5C). Additionally, there was a trend toward a decrease in cell perimeter (mean difference 228.57; 95% CI −53.637, 510.781; *p* = 0.087; Figure 5D), although no statistically significant differences were observed compared to those in the control group. However, circularity (mean difference − 0.01; 95% CI −0.041, 0.011; *p* = 0.189; Figure 5E), solidity (mean difference − 0.03; 95% CI −0.103, 0.041; *p* = 0.298; Figure 5F), and aspect ratio (mean difference − 0.019; 95% CI −0.465, 0.094; *p* = 0.139; Figure 5G) did not exhibit any statistically significant differences between the treatments. On the other hand, the Sholl analysis (Figure 6A) indicated a trend toward a reduction in the number of ramifications in the LPS-treated group compared to the control group (Figure 6B). Nevertheless, the AUC analysis derived from the Sholl curves did not reveal statistically significant differences in the number of ramifications between the two groups (mean difference 71.36; 95% CI 0, 155.74; *p* = 0.078; Figure 6C).

### 3.3 LPS-mediated chronic TLR4 stimulation did not affect the number of tau inclusions

**Figure 7:**
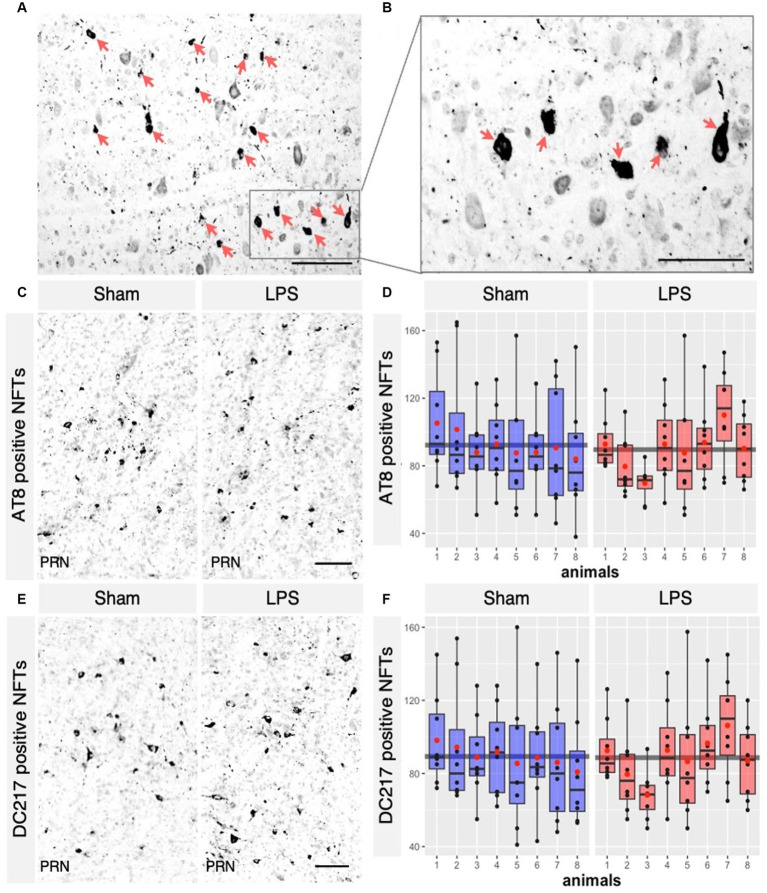
LPS-mediated chronic TLR4 stimulation had no effect on the tangle load observed across the groups. **(A)** AT8 positive Neurofibrillary tangles (NFTs) in the brainstem of R3m4 mice, **(B)** in higher magnification that were detected (pointed with red arrows). **(C)** Representative IHC sections of the brainstem probed for AT8 in both the sham and LPS-treated groups and for DC217 in both the sham and LPS-treated **(E)** groups. **(D,F)** Quantification of the NFTs demonstrated no significant changes in the numbers of AT8 positive NFTs (*p* > 0.05) or DC217 (*p* > 0.05) in the LPS- and sham-treated group. Boxes indicate medians and quartiles for morphometric parameters per animal; whiskers indicate quartiles ± 1.5× IQR. The black dots indicate the tangle count per slice. The red dots indicate the arithmetic mean. Magnification, 40×, 60×, 20×. Scale bar, 100 μm.

We next investigated whether chronic neuroinflammation could modify the number of tau tangles bearing neurons in the R3m4-transgenic mouse model. We quantified only neurons with apparent fibrillary inclusions (Figures 7A,B). A total NFT count was performed using the monoclonal antibodies AT8 (phospho-tau Ser202/Thr205) and DC217 (phospho-tau Thr217) within the selected sections of the brainstem. AT8 serves as a golden standard for staining of tau pathology in human brains (Alafuzoff et al., 2008), while p-tau Thr217 is considered the best available biomarker for AD (Ashton et al., 2024). We found no significant difference in the tangle-bearing neuron count between the LPS-treated group and the sham-treated group stained with AT8 (mean difference 2.63; 95% CI −7.856, 13.118; *p* = 0.599; Figures 7C,D) or DC217 (mean difference 0.63; 95% CI −8.761, 10.031; *p* = 0.8939; Figures 7E,F). We did not observe any significant difference between the sexes in sham treated with AT8 (*p* = 0.43) or DC217 (*p* = 0.65) and LPS-treated animals with AT8 (*p* = 0.11) or DC217 (*p* = 0.23).

**Figure 8:**
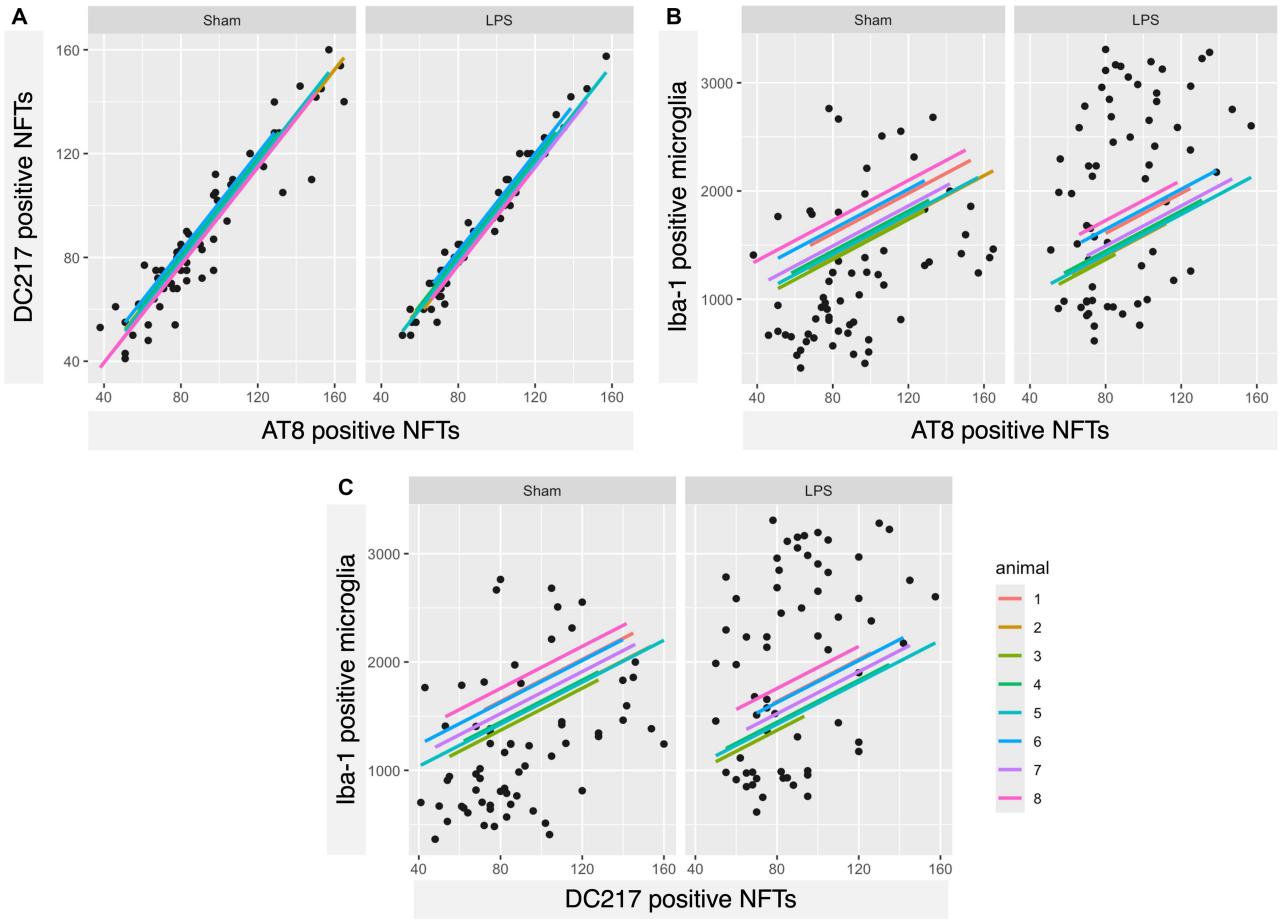
A strong positive correlation was observed between AT8- and DC217-positive tau tangles and each other and, interestingly, with microglial count. **(A)** Scatterplots with regression lines for each animal demonstrating that there was a positive correlation between DC217 and AT8-positive tau tangles in both sham (*p* < 0.00001)- and LPS (*p* < 0.00001)-treated animals; **(B)** AT8-positive tau tangles with Iba-1^+^ microglia in the sham (*p* = 0.00121)- and LPS-treated (*p* = 0.00143) groups; **(C)** DC217-positive tau tangles with Iba-1^+^ microglia in the sham (*p* = 0.00188) and LPS-treated (*p* = 0.00223) groups.

We found a significant correlation between the number of AT8- and DC217-positive tangles in both sham (rho = 0.93, 95% CI 0.9019, 0.9629, *p* < 0.00001) and LPS-treated (rho = 0.983, 95% CI 0.9727, 0.9899, *p* < 0.00001) animals (Figure 8A). Interestingly, the number of microglia correlated well with the number of AT8-positive tangles in the sham (rho = 0.392, 95% CI 0.1621, 0.5819, *p* = 0.00121) and LPS-treated (rho = 0.387, 95% CI 0.1560, 0.5778, *p* = 0.00143) group (Figure 8B) and DC217-positive tangles in the sham (rho = 0.378, 95% CI 0.1459, 0.5709, *p* = 0.00188) and LPS-treated (rho = 0.373, 95% CI 0.1397, 0.5666, *p* = 0.00223) groups (Figure 8C). In the sham group, there were, on average, 13 microglia per AT8-positive tangles (AT8 mean = 92.22; Iba-1 mean = 1240.28), while in the LPS-treated group, there were 22 microglia per tangle (AT8 mean = 89.59; Iba-1 mean = 1986.41), indicating a 1.7-fold increase. Similarly, the sham group had 14 microglia per DC217positive tangle (DC217 mean = 89.29; Iba-1 mean = 1240.28), and the LPS-treated group had 21 microglia per tangle (DC217 mean = 88.65; Iba-1 mean = 1986.41), indicating a 1.5-fold increase in microglial presence in the respective experimental groups.

### 3.4 LPS-mediated chronic TLR4 stimulation led to a significant reduction in phosphorylated tau in the hippocampus but not in the brainstem

**Figure 9:**
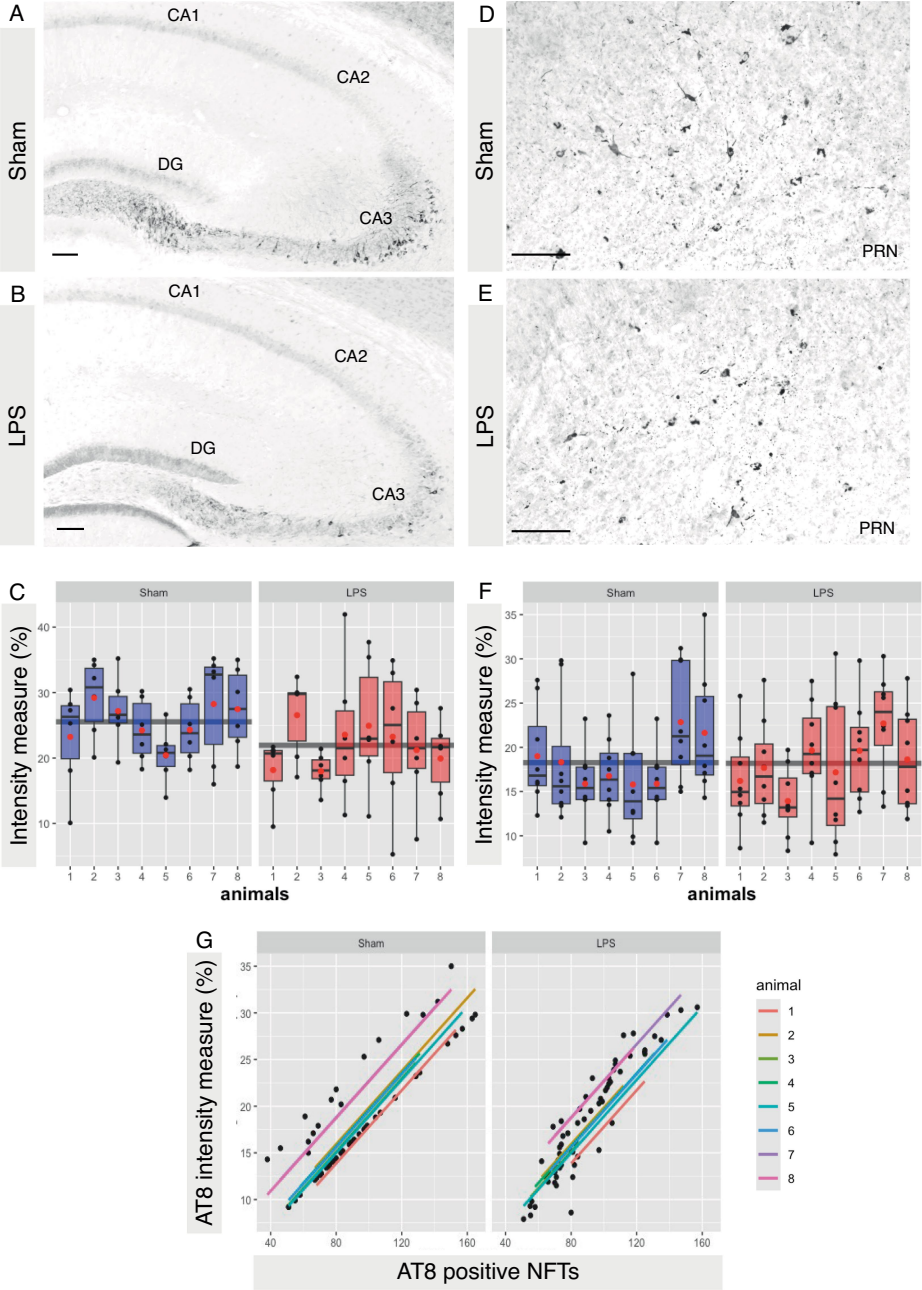
LPS-mediated chronic TLR4 stimulation led to a reduction in tau phosphorylation. **(A,B)** Representative image demonstrating abnormally phosphorylated tau within the somatodendritic compartment of pyramidal neurons in the CA3 subfield of the hippocampus, characterized by a diffuse signal that was more pronounced in sham-treated animals than in LPS-treated animals. **(C)** A significant reduction in tau phosphorylation was observed in the hippocampus (*p* < 0.05). **(D,E)** Representative image demonstrating abnormally phosphorylated tau and tangles bearing neurons in the brainstem. **(F)** No significant change in the brainstem was observed (*p* > 0.5). **(G)** Scatterplots with regression lines for each animal demonstrating that there was a positive correlation between AT8 signal intensity and AT8-positive tau tangles in both sham (*p* < 0.00001)- and LPS-treated (*p* < 0.00001) animals. Boxes indicate medians and quartiles for AT8-positive neurons per animal; whiskers indicate quartiles ± 1.5× IQR. The black dots indicate AT8-positive neurons per slice. The red dots indicate the arithmetic mean. Magnification, 20×. Scale bar, 100 μm.

Finally, we aimed to investigate whether chronic neuroinflammation, simulated via LPS administration, could induce tau hyperphosphorylation in R3m4 mice. We quantified signal intensity across the entire hippocampal formation. It is to be noted here that, despite the expression of truncated tau, R3m4 mice do not develop tau tangle pathology in the hippocampus (Zimova et al., 2016). However, in the sham group, we identified abnormally phosphorylated tau in the somatodendritic compartment of CA3 pyramidal neurons, presenting as a diffuse signal (Figures 9A,B). Compared to the sham group, the LPS-treated group exhibited significantly reduced levels of tau hyperphosphorylation in the hippocampus (mean difference 21.95; 95% CI 19.631, 24.267; *p* < 0.0341; Figure 9C). To assess if similar changes occurred in the brainstem—the brain area affected by NFTs, we measured signal intensity across brainstem slices and compared the sham and LPS-treated groups (Figures 9D,E). Our analysis revealed no significant change in tau phosphorylation levels in the brainstem (mean difference 0.07; 95% CI −2.806, 2.948; *p* < 0.9584; Figure 9F). Notably, there was a significant correlation between the levels of phosphorylation and the number of tangles in sham (r = 0.876, *p* < 0.00001) and LPS-treated (r = 0.936, *p* < 0.00001) group (Figure 9G).

## 4 Discussion

Glial-mediated neuroinflammation stands out as a shared hallmark among AD and related tauopathies (Odfalk et al., 2022). Where microglia wield a double-edged sword, engaging in a myriad of intricate protective, as well as deleterious functions (Kinney et al., 2018; Leng and Edison, 2021; Andreasson et al., 2016). These intricate functions are regulated by several innate immune modulators, among which the family of Toll-like receptors (TLRs) acts as a key determinant influencing susceptibility or resilience to the progression of tauopathies (Momtazmanesh et al., 2020). In this study, we induced chronic systemic inflammation by activating the TLR4 signaling pathway with LPS, a common pathogen-associated molecular pattern (PAMP). This inflammation, characterized by reactive microgliosis, leads to tau hyperphosphorylation in the CNS via bloodstream inflammatory mediators (Lykhmus et al., 2016). Additionally, LPS also primes nod-like receptor pyrin domain-containing protein 3 (NLRP3) inflammasome, elevating pro-interleukin-1β (pro-IL-1β) and NLRP3 levels (Hornung and Latz, 2010). Interestingly, tau can also activate the NLRP3 inflammasome in a similar fashion (Stancu et al., 2019). Knocking out NLRP3 in tau transgenic tau22 mice is shown to reduce tau deposition and memory loss which was associated with a reduction in the levels of cleaved-caspase-1 and IL-1β (Ising et al., 2019). Despite this evidence, a direct causal link between chronic LPS-induced TLR-4 stimulation and these effects remains unclear, hindering clinical translation.

Here we demonstrated that the TLR4-mediated chronic systemic inflammation had no effect on the formation of tangles in mouse model expressing sporadic truncation of tau native to AD, despite persistent microglial reactivity. In contrast, prior studies employed either biweekly i.p. LPS administration at a lower dose (0.5 mg/kg) for 6 weeks in amyloid- and tau-transgenic 3 × Tg-AD mice (Joshi et al., 2014; Kitazawa et al., 2005; Sy et al., 2011) or a similar weekly i.p. LPS administration but at a much lower dose (0.15 mg/kg) for 12 weeks in tau-transgenic PS19 mice (Qin et al., 2016), our experimental design employed chronic LPS preconditioning with weekly i.p. administration of a high dose (5 mg/kg) of LPS for 9 weeks followed by sacrifice 5 week later, to exclude the acute inflammatory effects of the last LPS insult. Considering the established lower sensitivity of mice to LPS than humans (Sauter and Wolfensberger, 1980) and rabbits (Redl et al., 1993), with a lethal dose ranging from 10 to 40 mg/kg (Tateda et al., 1996; Mahapatra et al., 2018), our selected dose was the highest possible dose without inducing sepsis which has been previously tested in acute inflammatory setups (Qin et al., 2007).

While we noted enhanced proliferation of microglia and significant retraction of processes in response to chronic LPS preconditioning, which are indicative of reactive microgliosis (Shankaran et al., 2007; Furube et al., 2015; Kloss et al., 2001; Kondo et al., 2011), the retraction of processes was evident from a significant reduction in cell area and a trend toward decreased cell perimeter values. Despite these changes, none of the other analysed shape descriptors (circularity, solidity, or aspect ratio) from the morphometric analysis showed differences in microglia between the groups. One potential explanation is that the overstimulation of microglia due to LPS, coupled with the developing pathology, as observed in tau-transgenic PS19 (van Olst et al., 2020), resulted in an “exhaustive phenotype” (Millet et al., 2024; Wendeln et al., 2018). This phenotype is also observed in aged and APOE4 genotype AD brains (Millet et al., 2024).

Interestingly, while microgliosis was evident, we observed no significant difference in the total tangle count between the groups. These results align with previous studies that reported no changes in insoluble tau levels (Joshi et al., 2014) or tangle count (Lee et al., 2010) upon chronic LPS challenge. Interestingly, we observed a significant correlation between the number of microglia per tangle in both the LPS- and the sham-treated group. However, despite the significant increase of 1.5–1.7-fold in the LPS-treated group, the presence of microglia did not lead to a reduction in the number of tangles. This finding aligns with prior studies indicating minimal tau internalization by microglia in AD brains (Nilson et al., 2017; Bolos et al., 2016; Bolos et al., 2017) and in transgenic animal models (Nilson et al., 2017; Clayton et al., 2021). This finding suggested that under inflammatory conditions, microglia may not be able to preferentially internalize tau.

Finally, our findings demonstrated a significant reduction in tau hyperphosphorylation in the hippocampus after a TLR4-mediated chronic LPS preconditioning in R3m4 mice. Conversely, such reduction was not observed in the brainstem. Despite the similar expression profiles of tau between the brainstem and hippocampus (Zimova et al., 2016), there are distinct differences in their phosphorylation patterns (Filipcik et al., 2012). This suggests that the specific tau phosphorylation signature in the hippocampus may confer protection against tangle formation (Schneider et al., 1999; Ittner et al., 2016; Strang et al., 2019). However, it is also unclear whether certain specific tau phospho-epitopes are added before or after the formation of tau inclusions, which may mitigate potential neuroprotective effects. Besides, R3m4 mouse model do not readily develop tangle pathology in the hippocampal formation during physiological aging or following chronic inflammation, as is observed here. However, robust tau tangle pathology is evident when intracerebral inoculation with human brain-derived AD-tau fibrils is performed.

Furthermore, the effect of LPS on tau phosphorylation remains controversial. While the reductions of tau phosphorylation were reported in the hippocampus of PS19 mice treated with TLR2-interaction domain of the MyD88 (wtTIDM) peptide (Dutta et al., 2023) and in 3 × Tg-AD mice treated with TLR9 agonists, short synthetic single-stranded DNA molecules containing unmethylated cytosine-guanosine oligodeoxynucleotides (CpG ODNs; Scholtzova et al., 2014). On the other hand, a dose-dependent increase in tau phosphorylation in 3 × Tg-AD mouse model was observed in other studies investigating chronic inflammation induced by LPS-mediated TLR4 activation (Joshi et al., 2014; Kitazawa et al., 2005; Sy et al., 2011). Interestingly, irrespective of whether TLR stimulation results in an increase or reduction in tau hyperphosphorylation, no discernible impact on tau tangle formation was noted in any of the abovementioned studies. These findings suggest that hyperphosphorylation alone may not be the exclusive factor driving tau tangle formation (Wegmann et al., 2021), characterizing the causal relationship between chronic inflammation-mediated tau phosphorylation and subsequent tangle formation as resembling a “distant analogy.”

## 5 Limitations

The selected model in this study exhibited tau pathology exclusively within the brainstem. The evaluation of behavioural and cognitive changes was omitted because the impact of LPS on cognition and behaviour has been comprehensively documented in previous studies. The short lifespan of this transgenic line presents a significant limitation, making it challenging to conduct analyses over extended and multiple meaningful time points. Additionally, no assessment of peripheral inflammation markers was conducted, again, given the well-established understanding of immune system activation via LPS.

## 6 Significance statement

This study demonstrates that chronic TLR4 stimulation via LPS-treatment does not exacerbate tau tangle pathology in a mouse model of sporadic AD, despite a significant increase in microglial number and an exhausted microglial phenotype. While we observed reduced tau phosphorylation levels in the hippocampus, no reduction in tau phosphorylation or tangles was noted in the brainstem, indicating that chronic TLR4 activation does not contribute to these processes. These findings offer new insights into the relationship between neuroinflammation and tau pathology, challenging the prevailing view that chronic inflammation uniformly exacerbates tau pathology in neurodegenerative diseases.

## Data Availability

The raw data supporting the conclusions of this article will be made available by the authors, without undue reservation.

## References

[ref1] Alafuzoff I. Arzberger T. Al-Sarraj S. Bodi I. Bogdanovic N. Braak H.. (2008). Staging of neurofibrillary pathology in Alzheimer's disease: a study of the BrainNet Europe consortium. Brain Pathol. 18, 484–496. doi: 10.1111/j.1750-3639.2008.00147.x, PMID: 18371174 PMC2659377

[ref2] Andreasson K. I. Bachstetter A. D. Colonna M. Ginhoux F. Holmes C. Lamb B.. (2016). Targeting innate immunity for neurodegenerative disorders of the central nervous system. J. Neurochem. 138, 653–693. doi: 10.1111/jnc.13667, PMID: 27248001 PMC5433264

[ref3] Ashton N. J. Brum W. S. Di Molfetta G. Benedet A. L. Arslan B. Jonaitis E.. (2024). Diagnostic accuracy of a plasma phosphorylated tau 217 immunoassay for Alzheimer disease pathology. JAMA Neurol. 81, 255–263. doi: 10.1001/jamaneurol.2023.5319, PMID: 38252443 PMC10804282

[ref4] Bakdash J. Z. Marusich L. R. (2017). Repeated measures correlation. Front. Psychol. 8:456. doi: 10.3389/fpsyg.2017.00456, PMID: 28439244 PMC5383908

[ref5] Bankhead P. Loughrey M. B. Fernandez J. A. Dombrowski Y. McArt D. G. Dunne P. D.. (2017). QuPath: open source software for digital pathology image analysis. Sci. Rep. 7:16878. doi: 10.1038/s41598-017-17204-5, PMID: 29203879 PMC5715110

[ref6] Barron M. Gartlon J. Dawson L. A. Atkinson P. J. Pardon M. C. (2017). A state of delirium: deciphering the effect of inflammation on tau pathology in Alzheimer's disease. Exp. Gerontol. 94, 103–107. doi: 10.1016/j.exger.2016.12.006, PMID: 27979768 PMC5479936

[ref7] Batista C. R. A. Gomes G. F. Candelario-Jalil E. Fiebich B. L. de Oliveira A. C. P. (2019). Lipopolysaccharide-induced neuroinflammation as a bridge to understand neurodegeneration. Int. J. Mol. Sci. 20:2293. doi: 10.3390/ijms20092293, PMID: 31075861 PMC6539529

[ref8] Beam A. Clinger E. Hao L. (2021). Effect of diet and dietary components on the composition of the gut microbiota. Nutrients 13:2795. doi: 10.3390/nu13082795, PMID: 34444955 PMC8398149

[ref9] Bellucci A. Bugiani O. Ghetti B. Spillantini M. G. (2011). Presence of reactive microglia and neuroinflammatory mediators in a case of frontotemporal dementia with P301S mutation. Neurodegener. Dis. 8, 221–229. doi: 10.1159/000322228, PMID: 21212632 PMC3214942

[ref10] Bolos M. Llorens-Martin M. Jurado-Arjona J. Hernandez F. Rabano A. Avila J. (2016). Direct evidence of internalization of tau by microglia in vitro and in vivo. J. Alzheimers Dis. 50, 77–87. doi: 10.3233/JAD-150704, PMID: 26638867

[ref11] Bolos M. Llorens-Martin M. Perea J. R. Jurado-Arjona J. Rabano A. Hernandez F.. (2017). Absence of CX3CR1 impairs the internalization of tau by microglia. Mol. Neurodegener. 12:59. doi: 10.1186/s13024-017-0200-1, PMID: 28810892 PMC5558740

[ref12] Cani P. D. Amar J. Iglesias M. A. Poggi M. Knauf C. Bastelica D.. (2007). Metabolic endotoxemia initiates obesity and insulin resistance. Diabetes 56, 1761–1772. doi: 10.2337/db06-1491, PMID: 17456850

[ref13] Catorce M. N. Gevorkian G. (2016). LPS-induced murine neuroinflammation model: main features and suitability for pre-clinical assessment of nutraceuticals. Curr. Neuropharmacol. 14, 155–164. doi: 10.2174/1570159X14666151204122017, PMID: 26639457 PMC4825946

[ref14] Clayton K. Delpech J. C. Herron S. Iwahara N. Ericsson M. Saito T.. (2021). Plaque associated microglia hyper-secrete extracellular vesicles and accelerate tau propagation in a humanized APP mouse model. Mol. Neurodegener. 16:18. doi: 10.1186/s13024-021-00440-9, PMID: 33752701 PMC7986521

[ref15] Dutta D. Jana M. Paidi R. K. Majumder M. Raha S. Dasarathy S.. (2023). Tau fibrils induce glial inflammation and neuropathology via TLR2 in Alzheimer's disease-related mouse models. J. Clin. Invest. 133:e161987. doi: 10.1172/JCI161987, PMID: 37552543 PMC10503811

[ref16] Eikelenboom P. van Exel E. Hoozemans J. J. Veerhuis R. Rozemuller A. J. van Gool W. A. (2010). Neuroinflammation - an early event in both the history and pathogenesis of Alzheimer's disease. Neurodegener. Dis. 7, 38–41. doi: 10.1159/000283480, PMID: 20160456

[ref17] Fernandez-Arjona M. D. M. Grondona J. M. Granados-Duran P. Fernandez-Llebrez P. Lopez-Avalos M. D. (2017). Microglia morphological categorization in a rat model of neuroinflammation by hierarchical cluster and principal components analysis. Front. Cell. Neurosci. 11:235. doi: 10.3389/fncel.2017.00235, PMID: 28848398 PMC5550745

[ref18] Filipcik P. Zilka N. Bugos O. Kucerak J. Koson P. Novak P.. (2012). First transgenic rat model developing progressive cortical neurofibrillary tangles. Neurobiol. Aging 33, 1448–1456. doi: 10.1016/j.neurobiolaging.2010.10.015, PMID: 21196063

[ref19] Furube E. Morita M. Miyata S. (2015). Characterization of neural stem cells and their progeny in the sensory circumventricular organs of adult mouse. Cell Tissue Res. 362, 347–365. doi: 10.1007/s00441-015-2201-0, PMID: 25994374

[ref20] Grubman A. Chew G. Ouyang J. F. Sun G. Choo X. Y. McLean C.. (2019). A single-cell atlas of entorhinal cortex from individuals with Alzheimer's disease reveals cell-type-specific gene expression regulation. Nat. Neurosci. 22, 2087–2097. doi: 10.1038/s41593-019-0539-4, PMID: 31768052

[ref21] Grundke-Iqbal I. Iqbal K. Tung Y. C. Quinlan M. Wisniewski H. M. Binder L. I. (1986). Abnormal phosphorylation of the microtubule-associated protein tau (tau) in Alzheimer cytoskeletal pathology. Proc. Natl. Acad. Sci. USA 83, 4913–4917. doi: 10.1073/pnas.83.13.4913, PMID: 3088567 PMC323854

[ref22] Hornung V. Latz E. (2010). Critical functions of priming and lysosomal damage for NLRP3 activation. Eur. J. Immunol. 40, 620–623. doi: 10.1002/eji.200940185, PMID: 20201015 PMC3893565

[ref23] Ishizawa K. Dickson D. W. (2001). Microglial activation parallels system degeneration in progressive supranuclear palsy and corticobasal degeneration. J. Neuropathol. Exp. Neurol. 60, 647–657. doi: 10.1093/jnen/60.6.647, PMID: 11398841

[ref24] Ising C. Venegas C. Zhang S. Scheiblich H. Schmidt S. V. Vieira-Saecker A.. (2019). NLRP3 inflammasome activation drives tau pathology. Nature 575, 669–673. doi: 10.1038/s41586-019-1769-z, PMID: 31748742 PMC7324015

[ref25] Ittner A. Chua S. W. Bertz J. Volkerling A. van der Hoven J. Gladbach A.. (2016). Site-specific phosphorylation of tau inhibits amyloid-beta toxicity in Alzheimer's mice. Science 354, 904–908. doi: 10.1126/science.aah6205, PMID: 27856911

[ref26] Jaworski T. Lechat B. Demedts D. Gielis L. Devijver H. Borghgraef P.. (2011). Dendritic degeneration, neurovascular defects, and inflammation precede neuronal loss in a mouse model for tau-mediated neurodegeneration. Am. J. Pathol. 179, 2001–2015. doi: 10.1016/j.ajpath.2011.06.025, PMID: 21839061 PMC3181369

[ref27] Jayashree B. Bibin Y. S. Prabhu D. Shanthirani C. S. Gokulakrishnan K. Lakshmi B. S.. (2014). Increased circulatory levels of lipopolysaccharide (LPS) and zonulin signify novel biomarkers of proinflammation in patients with type 2 diabetes. Mol. Cell. Biochem. 388, 203–210. doi: 10.1007/s11010-013-1911-4, PMID: 24347174

[ref28] Joshi Y. B. Giannopoulos P. F. Chu J. Pratico D. (2014). Modulation of lipopolysaccharide-induced memory insult, gamma-secretase, and neuroinflammation in triple transgenic mice by 5-lipoxygenase. Neurobiol. Aging 35, 1024–1031. doi: 10.1016/j.neurobiolaging.2013.11.016, PMID: 24332986 PMC3948206

[ref29] Keren-Shaul H. Spinrad A. Weiner A. Matcovitch-Natan O. Dvir-Szternfeld R. Ulland T. K.. (2017). A unique microglia type associated with restricting development of Alzheimer's disease. Cell 169, 1276–1290.e17. doi: 10.1016/j.cell.2017.05.018 28602351

[ref30] Kinney J. W. Bemiller S. M. Murtishaw A. S. Leisgang A. M. Salazar A. M. Lamb B. T. (2018). Inflammation as a central mechanism in Alzheimer's disease. Alzheimers Dement. (N Y) 4, 575–590. doi: 10.1016/j.trci.2018.06.014, PMID: 30406177 PMC6214864

[ref31] Kitazawa M. Oddo S. Yamasaki T. R. Green K. N. LaFerla F. M. (2005). Lipopolysaccharide-induced inflammation exacerbates tau pathology by a cyclin-dependent kinase 5-mediated pathway in a transgenic model of Alzheimer's disease. J. Neurosci. 25, 8843–8853. doi: 10.1523/JNEUROSCI.2868-05.2005, PMID: 16192374 PMC6725603

[ref32] Kloss C. U. Bohatschek M. Kreutzberg G. W. Raivich G. (2001). Effect of lipopolysaccharide on the morphology and integrin immunoreactivity of ramified microglia in the mouse brain and in cell culture. Exp. Neurol. 168, 32–46. doi: 10.1006/exnr.2000.7575, PMID: 11170719

[ref33] Kondo S. Kohsaka S. Okabe S. (2011). Long-term changes of spine dynamics and microglia after transient peripheral immune response triggered by LPS in vivo. Mol. Brain 4:27. doi: 10.1186/1756-6606-4-27, PMID: 21682853 PMC3138393

[ref34] Koson P. Zilka N. Kovac A. Kovacech B. Korenova M. Filipcik P.. (2008). Truncated tau expression levels determine life span of a rat model of tauopathy without causing neuronal loss or correlating with terminal neurofibrillary tangle load. Eur. J. Neurosci. 28, 239–246. doi: 10.1111/j.1460-9568.2008.06329.x, PMID: 18702695

[ref35] Laurent C. Buee L. Blum D. (2018). Tau and neuroinflammation: what impact for Alzheimer's disease and tauopathies? Biom. J. 41, 21–33. doi: 10.1016/j.bj.2018.01.003 PMC6138617 29673549

[ref36] Lee D. C. Rizer J. Selenica M. L. Reid P. Kraft C. Johnson A.. (2010). LPS- induced inflammation exacerbates phospho-tau pathology in rTg4510 mice. J. Neuroinflammation 7:56. doi: 10.1186/1742-2094-7-56, PMID: 20846376 PMC2949628

[ref37] Leng F. Edison P. (2021). Neuroinflammation and microglial activation in Alzheimer disease: where do we go from here? Nat. Rev. Neurol. 17, 157–172. doi: 10.1038/s41582-020-00435-y, PMID: 33318676

[ref38] Leyns C. E. G. Holtzman D. M. (2017). Glial contributions to neurodegeneration in tauopathies. Mol. Neurodegener. 12:50. doi: 10.1186/s13024-017-0192-x, PMID: 28662669 PMC5492997

[ref39] Liddelow S. A. Guttenplan K. A. Clarke L. E. Bennett F. C. Bohlen C. J. Schirmer L.. (2017). Neurotoxic reactive astrocytes are induced by activated microglia. Nature 541, 481–487. doi: 10.1038/nature21029, PMID: 28099414 PMC5404890

[ref40] López-González I. Schlüter A. Aso E. Garcia-Esparcia P. Ansoleaga B. LLorens F.. (2015). Neuroinflammatory signals in Alzheimer disease and APP/PS1 transgenic mice: correlations with plaques, tangles, and oligomeric species. J. Neuropathol. Exp. Neurol. 74, 319–344. doi: 10.1097/NEN.0000000000000176, PMID: 25756590

[ref41] Lykhmus O. Mishra N. Koval L. Kalashnyk O. Gergalova G. Uspenska K.. (2016). Molecular mechanisms regulating LPS-induced inflammation in the brain. Front. Mol. Neurosci. 9:19. doi: 10.3389/fnmol.2016.00019, PMID: 27013966 PMC4781876

[ref42] Mahapatra S. Ying L. Ho P. P. Kurnellas M. Rothbard J. Steinman L.. (2018). An amyloidogenic hexapeptide derived from amylin attenuates inflammation and acute lung injury in murine sepsis. PLoS One 13:e0199206. doi: 10.1371/journal.pone.0199206, PMID: 29990318 PMC6039005

[ref43] Mate V. Smolek T. Kazmerova Z. V. Jadhav S. Brezovakova V. Jurkanin B.. (2022). Enriched environment ameliorates propagation of tau pathology and improves cognition in rat model of tauopathy. Front. Aging Neurosci. 14:935973. doi: 10.3389/fnagi.2022.935973, PMID: 35966785 PMC9363241

[ref44] Mathys H. Davila-Velderrain J. Peng Z. Gao F. Mohammadi S. Young J. Z.. (2019). Single-cell transcriptomic analysis of Alzheimer's disease. Nature 570, 332–337. doi: 10.1038/s41586-019-1195-2, PMID: 31042697 PMC6865822

[ref45] McMurray L. Macdonald J. A. Ramakrishnan N. K. Zhao Y. Williamson D. W. Tietz O.. (2021). Synthesis and assessment of novel probes for imaging tau pathology in transgenic mouse and rat models. ACS Chem. Neurosci. 12, 1885–1893. doi: 10.1021/acschemneuro.0c00790, PMID: 33689290 PMC8176454

[ref46] Millet A. Ledo J. H. Tavazoie S. F. (2024). An exhausted-like microglial population accumulates in aged and APOE4 genotype Alzheimer's brains. Immunity 57, 153–170.e6. doi: 10.1016/j.immuni.2023.12.001, PMID: 38159571 PMC10805152

[ref47] Mohammad S. Thiemermann C. (2020). Role of metabolic endotoxemia in systemic inflammation and potential interventions. Front. Immunol. 11:594150. doi: 10.3389/fimmu.2020.594150, PMID: 33505393 PMC7829348

[ref48] Momtazmanesh S. Perry G. Rezaei N. (2020). Toll-like receptors in Alzheimer's disease. J. Neuroimmunol. 348:577362. doi: 10.1016/j.jneuroim.2020.577362, PMID: 32858428

[ref49] Nilson A. N. English K. C. Gerson J. E. Barton Whittle T. Nicolas Crain C. Xue J.. (2017). Tau oligomers associate with inflammation in the brain and retina of tauopathy mice and in neurodegenerative diseases. J. Alzheimers Dis. 55, 1083–1099. doi: 10.3233/JAD-160912, PMID: 27716675 PMC5147514

[ref50] Novak M. Kabat J. Wischik C. M. (1993). Molecular characterization of the minimal protease resistant tau unit of the Alzheimer's disease paired helical filament. EMBO J. 12, 365–370. doi: 10.1002/j.1460-2075.1993.tb05665.x, PMID: 7679073 PMC413214

[ref51] Odfalk K. F. Bieniek K. F. Hopp S. C. (2022). Microglia: friend and foe in tauopathy. Prog. Neurobiol. 216:102306. doi: 10.1016/j.pneurobio.2022.102306, PMID: 35714860 PMC9378545

[ref52] Pinheiro J. C. Bates D. M. (2006). Mixed-effects models in S and S-PLUS. New York, NY: Springer.

[ref53] Qin Y. Liu Y. Hao W. Decker Y. Tomic I. Menger M. D.. (2016). Stimulation of TLR4 attenuates Alzheimer's disease-related symptoms and pathology in tau-transgenic mice. J. Immunol. 197, 3281–3292. doi: 10.4049/jimmunol.1600873, PMID: 27605009

[ref54] Qin L. Wu X. Block M. L. Liu Y. Breese G. R. Hong J. S.. (2007). Systemic LPS causes chronic neuroinflammation and progressive neurodegeneration. Glia 55, 453–462. doi: 10.1002/glia.20467, PMID: 17203472 PMC2871685

[ref55] R Development Core Team (2023). R: a language and environment for statistical computing. R Foundation for Statistical Computing, Vienna, Austria. R Foundation for Staitsical Computing.

[ref56] Redl H. Bahrami S. Schlag G. Traber D. L. (1993). Clinical detection of LPS and animal models of endotoxemia. Immunobiology 187, 330–345. doi: 10.1016/S0171-2985(11)80348-7, PMID: 8330902

[ref57] Sanchez-Tapia M. Mimenza-Alvarado A. Granados-Dominguez L. Flores-Lopez A. Lopez-Barradas A. Ortiz V.. (2023). The gut microbiota-brain Axis during aging, mild cognitive impairment and dementia: role of tau protein, beta-amyloid and LPS in serum and curli protein in stool. Nutrients 15:932. doi: 10.3390/nu15040932, PMID: 36839291 PMC9961602

[ref58] Sasaki A. Kawarabayashi T. Murakami T. Matsubara E. Ikeda M. Hagiwara H.. (2008). Microglial activation in brain lesions with tau deposits: comparison of human tauopathies and tau transgenic mice TgTauP301L. Brain Res. 1214, 159–168. doi: 10.1016/j.brainres.2008.02.084, PMID: 18457819

[ref59] Sauter C. Wolfensberger C. (1980). Interferon in human serum after injection of endotoxin. Lancet 2, 852–853. doi: 10.1016/s0140-6736(80)90189-0, PMID: 6159510

[ref60] Schneider A. Biernat J. von Bergen M. Mandelkow E. Mandelkow E. M. (1999). Phosphorylation that detaches tau protein from microtubules (Ser262, Ser214) also protects it against aggregation into Alzheimer paired helical filaments. Biochemistry 38, 3549–3558. doi: 10.1021/bi981874p, PMID: 10090741

[ref61] Scholtzova H. Chianchiano P. Pan J. Sun Y. Goni F. Mehta P. D.. (2014). Amyloid beta and tau Alzheimer's disease related pathology is reduced by toll-like receptor 9 stimulation. Acta Neuropathol. Commun. 2:101. doi: 10.1186/s40478-014-0101-2, PMID: 25178404 PMC4171548

[ref62] Seok J. Warren H. S. Cuenca A. G. Mindrinos M. N. Baker H. V. Xu W.. (2013). Genomic responses in mouse models poorly mimic human inflammatory diseases. Proc. Natl. Acad. Sci. USA 110, 3507–3512. doi: 10.1073/pnas.1222878110, PMID: 23401516 PMC3587220

[ref63] Serrano-Pozo A. Gomez-Isla T. Growdon J. H. Frosch M. P. Hyman B. T. (2013). A phenotypic change but not proliferation underlies glial responses in Alzheimer disease. Am. J. Pathol. 182, 2332–2344. doi: 10.1016/j.ajpath.2013.02.031, PMID: 23602650 PMC3668030

[ref64] Shankaran M. Marino M. E. Busch R. Keim C. King C. Lee J.. (2007). Measurement of brain microglial proliferation rates in vivo in response to neuroinflammatory stimuli: application to drug discovery. J. Neurosci. Res. 85, 2374–2384. doi: 10.1002/jnr.21389, PMID: 17551981

[ref65] Simons M. Levin J. Dichgans M. (2023). Tipping points in neurodegeneration. Neuron 111, 2954–2968. doi: 10.1016/j.neuron.2023.05.031, PMID: 37385247

[ref66] Sobue A. Komine O. Hara Y. Endo F. Mizoguchi H. Watanabe S.. (2021). Microglial gene signature reveals loss of homeostatic microglia associated with neurodegeneration of Alzheimer's disease. Acta Neuropathol. Commun. 9:1. doi: 10.1186/s40478-020-01099-x, PMID: 33402227 PMC7786928

[ref67] Stancu I. C. Cremers N. Vanrusselt H. Couturier J. Vanoosthuyse A. Kessels S.. (2019). Aggregated tau activates NLRP3-ASC inflammasome exacerbating exogenously seeded and non-exogenously seeded tau pathology in vivo. Acta Neuropathol. 137, 599–617. doi: 10.1007/s00401-018-01957-y, PMID: 30721409 PMC6426830

[ref68] Strang K. H. Sorrentino Z. A. Riffe C. J. Gorion K. M. Vijayaraghavan N. Golde T. E.. (2019). Phosphorylation of serine 305 in tau inhibits aggregation. Neurosci. Lett. 692, 187–192. doi: 10.1016/j.neulet.2018.11.011, PMID: 30423399 PMC6351168

[ref69] Sy M. Kitazawa M. Medeiros R. Whitman L. Cheng D. Lane T. E.. (2011). Inflammation induced by infection potentiates tau pathological features in transgenic mice. Am. J. Pathol. 178, 2811–2822. doi: 10.1016/j.ajpath.2011.02.012, PMID: 21531375 PMC3124234

[ref70] Tateda K. Matsumoto T. Miyazaki S. Yamaguchi K. (1996). Lipopolysaccharide-induced lethality and cytokine production in aged mice. Infect. Immun. 64, 769–774. doi: 10.1128/iai.64.3.769-774.1996, PMID: 8641780 PMC173836

[ref71] Tukey J. W. (1962). The future of data analysis. Ann. Math. Stat. 33, 1–67. doi: 10.1214/aoms/1177704711, PMID: 39193763

[ref72] van Olst L. Verhaege D. Franssen M. Kamermans A. Roucourt B. Carmans S.. (2020). Microglial activation arises after aggregation of phosphorylated-tau in a neuron-specific P301S tauopathy mouse model. Neurobiol. Aging 89, 89–98. doi: 10.1016/j.neurobiolaging.2020.01.003, PMID: 32008854

[ref73] Wang J. Z. Xia Y. Y. Grundke-Iqbal I. Iqbal K. (2013). Abnormal hyperphosphorylation of tau: sites, regulation, and molecular mechanism of neurofibrillary degeneration. J. Alzheimers Dis. 33, S123–S139. 22710920 10.3233/JAD-2012-129031

[ref74] Wegmann S. Biernat J. Mandelkow E. (2021). A current view on tau protein phosphorylation in Alzheimer's disease. Curr. Opin. Neurobiol. 69, 131–138. doi: 10.1016/j.conb.2021.03.003, PMID: 33892381

[ref75] Wendeln A. C. Degenhardt K. Kaurani L. Gertig M. Ulas T. Jain G.. (2018). Innate immune memory in the brain shapes neurological disease hallmarks. Nature 556, 332–338. doi: 10.1038/s41586-018-0023-4, PMID: 29643512 PMC6038912

[ref76] Wiedermann C. J. Kiechl S. Dunzendorfer S. Schratzberger P. Egger G. Oberhollenzer F.. (1999). Association of endotoxemia with carotid atherosclerosis and cardiovascular disease: prospective results from the Bruneck study. J. Am. Coll. Cardiol. 34, 1975–1981. doi: 10.1016/S0735-1097(99)00448-9, PMID: 10588212

[ref77] Yoshiyama Y. Higuchi M. Zhang B. Huang S. M. Iwata N. Saido T. C.. (2007). Synapse loss and microglial activation precede tangles in a P301S tauopathy mouse model. Neuron 53, 337–351. doi: 10.1016/j.neuron.2007.01.010, PMID: 17270732

[ref78] Young K. Morrison H. (2018). Quantifying microglia morphology from photomicrographs of immunohistochemistry prepared tissue using ImageJ. J. Vis. Exp. 136:57648. doi: 10.3791/57648-v, PMID: 29939190 PMC6103256

[ref79] Zhou Y. Song W. M. Andhey P. S. Swain A. Levy T. Miller K. R.. (2020). Human and mouse single-nucleus transcriptomics reveal TREM2-dependent and TREM2-independent cellular responses in Alzheimer's disease. Nat. Med. 26, 131–142. doi: 10.1038/s41591-019-0695-9, PMID: 31932797 PMC6980793

[ref80] Zilka N. Kazmerova Z. Jadhav S. Neradil P. Madari A. Obetkova D.. (2012). Who fans the flames of Alzheimer's disease brains? Misfolded tau on the crossroad of neurodegenerative and inflammatory pathways. J. Neuroinflammation 9:47. 22397366 10.1186/1742-2094-9-47 PMC3334709

[ref81] Zimova I. Brezovakova V. Hromadka T. Weisova P. Cubinkova V. Valachova B.. (2016). Human truncated tau induces mature neurofibrillary pathology in a mouse model of human tauopathy. J. Alzheimers Dis. 54, 831–843. doi: 10.3233/JAD-160347, PMID: 27567836

